# Worldwide Niche and Future Potential Distribution of *Culicoides imicola*, a Major Vector of Bluetongue and African Horse Sickness Viruses

**DOI:** 10.1371/journal.pone.0112491

**Published:** 2014-11-12

**Authors:** Sylvain Guichard, Hélène Guis, Annelise Tran, Claire Garros, Thomas Balenghien, Darren J. Kriticos

**Affiliations:** 1 Cirad, UR AGIRs, F-34398, Montpellier, France; 2 InSTePP, University of Minnesota, St. Paul, MN, United States of America; 3 Cirad, UMR CMAEE, F-34398, Montpellier, France; 4 INRA, UMR1309 CMAEE, F-34398, Montpellier, France; 5 Cirad, UMR15 TETIS, F-34398, Montpellier, France; 6 CSIRO Agriculture Flagship and Biosecurity Flagship, GPO Box 1700, Canberra, ACT 2601, Australia; Onderstepoort Veterinary Institute, South Africa

## Abstract

We modelled the ecoclimatic niche of *Culicoides imicola*, a major arthropod vector of midge-borne viral pathogens affecting ruminants and equids, at fine scale and on a global extent, so as to provide insight into current and future risks of disease epizootics, and increase current knowledge of the species' ecology. Based on the known distribution and ecology of *C. imicola*, the species' response to monthly climatic conditions was characterised using CLIMEX with 10′ spatial resolution climatic datasets. The species' climatic niche was projected worldwide and under future climatic scenarios. The validated model highlights the role of irrigation in supporting the occurrence of *C. imicola* in arid regions. In Europe, the modelled potential distribution of *C. imicola* extended further West than its reported distribution, raising questions regarding ongoing process of colonization and non-climatic habitat factors. The CLIMEX model highlighted similar ecological niches for *C. imicola* and the Australasian *C. brevitarsis* raising questions on biogeography and biosecurity. Under the climate change scenarios considered, its' modelled potential distribution could expand northward in the Northern hemisphere, whereas in Africa its range may contract in the future. The biosecurity risks from bluetongue and African horse sickness viruses need to be re-evaluated in regions where the vector's niche is suitable. Under a warmer climate, the risk of vector-borne epizootic pathogens such as bluetongue and African horse sickness viruses are likely to increase as the climate suitability for *C. imicola* shifts poleward, especially in Western Europe.

## Introduction

Bluetongue (BT) and African horse sickness (AHS) viruses are vector-borne, causing diseases affecting ruminants and equids respectively. Each of these diseases causes significant economic losses and animal deaths [1]–[3].

Knowledge of the potential geographical range of these diseases can inform national and regional pest risk assessments, assisting biosecurity agencies to manage the invasion risks better by focusing scarce surveillance and prevention resources to protect endangered regions and assets [4].

**Figure 1:**
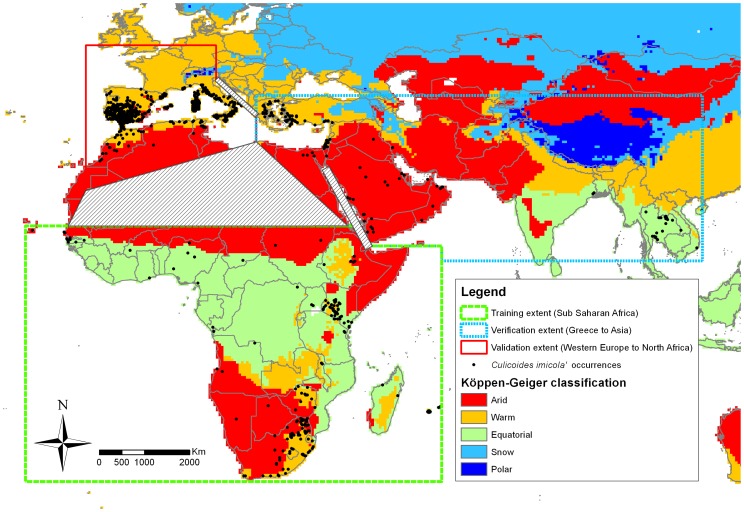
Distribution of *Culicoides imicola* and study area: *training*, *verification* and *validation* zones for modelling (with respectively 187, 131, 561 occurrences at 10′ granularity) with Köppen-Geiger climate simplified classification [33]. Cross-hatched areas are zones of inhospitable habitat (Adriatic Sea, Sahara Desert and Red Sea) separating the 3 model-fitting zones.

Several species of the genus *Culicoides* (Diptera: Ceratopogonidae) transmit BT, AHS and other major animal viruses. *Culicoides imicola*, an afro-oriental species, is widespread and has been reported from South Africa to southern Europe, and from western Africa to southern China (Figure 1 and Appendix S1 in File S1). This species has historically retained the interest because biological transmission of AHS and BT viruses by *Culicoides* was first demonstrated in this species, and to date, *C. imicola* remains the unique vector for both AHS and BT viruses in the afro-oriental region [5]–[7] whereas it has been suspected for other Afrotropical *Culicoides*, such as *C. bolitinos* among others [8]–[10]. It is also considered a key vector of both viruses in Africa, in the Middle East and the southern part of Europe due to the huge abundances it can attain [6].

The distribution of *C. imicola*, especially in southern Europe, is an old and actual subject of interest as illustrated by AHS and BT history: AHS is endemic in areas of Sub-Saharan Africa, as highlighted by the recent outbreaks in Senegal in 2007 [1], and has sporadically caused devastating outbreaks outside Africa, *eg.* in 1959–61 in the Middle East and in Asia (where over 300 000 equids died), and in 1987–90 on the Iberian Peninsula [7]. Less than 10 years later, several serotypes of BT virus were transmitted intensively, leading to the death of hundreds of thousands sheep in the Mediterranean basin, in areas never previously affected by the virus [11]. Abundant populations of *C. imicola* were recorded in most of these areas, where it was thought to be absent [11]. Some authors have suggested that the distribution of *C. imicola* has expanded its range northwards in the last decades in Mediterranean Europe as climatic changes reduced the frequency and intensity of cold temperatures that limit overwintering potential [12], [13], whereas other studies challenged this hypothesis [14], [15]. Under future climate conditions, Acevedo and co-authors projected that the distribution of *C. imicola* is expected to remain constant in Spain while its abundance is expected to increase due to increasing precipitation and changes in seasonality [16].

Studies of the regional climatic niche of *C. imicola* had been developed from its distribution in Spain and Portugal [17], Morocco [18], Italy [19], the Mediterranean [20], [21] and South Africa [22] using correlative modelling methods. However, our study is the first to model the ecoclimatic niche of *C. imicola*, based on its global distribution.

It is generally well accepted that our climate is changing in response to anthropogenic emissions of CO_2_ and other greenhouse gases. What is generally poorly appreciated is that our ability to forecast the future climatic conditions may be as limited by our ability to forecast factors such as patterns of socio-economic and technological development and human demography than by our ability to model the resulting atmospheric dynamics that drive global and regional climatic patterns [23]–[26]. These anthropogenic factors are difficult or impossible to predict at useful periods into the future, and they strongly influence the rate of emission of the greenhouse gases that are driving the observed and projected climatic changes. To address this problem the Intergovernmental Panel on Climate Change (IPCC) generated a series of social, economic and technological developmental storylines as a basis for the creation of future climate scenarios [27]. These storylines were intended to describe equally plausible future development paths that could be used to explore the sensitivity of climate-sensitive systems. Unfortunately, there has been a tendency to misunderstand or misuse the results of analyses based on these storylines, treating them as if they had predictive power. In this paper we apply a set of two scenarios to a biophysical model in order to highlight areas of potential future concerns for biosecurity risk managers. Note however, that in no way do we present these results as prediction of what will occur. Rather, they form a stress-test of the biosecurity risk patterns based on modelling using historical climatic data.

Ecoclimatic niche modelling has been used widely to explore the effects of climate change on the potential geographical range of many species of pest and conservation concern. CLIMEX [28] is a process-oriented niche model that can draw on both inferential and deductive modelling methods [29]. The model describes species' responses to climatic conditions and estimates species' potential distribution based upon climatic suitability. Due to its process-oriented formulation, CLIMEX is well suited for projecting species' potential distributions in novel climates such as inter-continental projections and future climatic scenarios [30]–[32].

As a primary step for evaluating the risks for these diseases at a particular location we investigated the ecoclimatic niche of *C. imicola*, a competent vector species for major animal viral diseases and projected its potential distribution under selected climate change scenarios.

## Methods

### Geographical distribution of *Culicoides imicola*

We compiled a geo-database of 1 381 individual presence records of *C. imicola* from published sources (Appendix S1 in File S1). To address problems with duplication and auto-correlation in assessing the goodness of fit, the individual point locations were transformed into 879 unique cells with 10′ resolution. The 879 presence records of *C. imicola* were split into three subsets (Figure 1): 1) a *training* dataset to develop the model (Sub-Saharan Africa, 187 records), 2) a *verification* dataset, to adjust the model at unfitted locations (South-Eastern Europe, Middle East and Asia, 131 records) and 3) a *validation* dataset used to infer the strengths and limitations of the model (Western Europe and North Africa, 561 records). In selecting the distribution data sub-sets, we aimed to keep a maximum range of different climate categories in each zone, but also independence between zones with physical boundaries (Saharan desert, Red Sea and Adriatic Sea, cross-hatched areas in Figure 1). We used the Köppen-Geiger (KG) climate classification [33] and ensured that *training*, *verification* and *validation* zones shared similar climatic ranges, except for an equatorial climate, which was not represented in the validation zone. Africa was assigned to training because *C. imicola* was first described there (holotype from Kenya, Kieffer, 1913 cited in Borkent, [34]) and is considered as the historical distribution area [35]. The validation dataset encompassed western Europe where the range of *C. imicola*'s distribution is still under debate [15], [36].

### Climate data

CLIMEX requires five climate variables: monthly values for minimum and maximum temperature, rainfall, and relative humidity at 9am and at 3pm. We used the CliMond 1975H_V1.1 fine spatial resolution (10′) global climate dataset [37]. CliMond includes monthly climate data averages centred on 1975, so-called historical climate dataset, and a total of eight future climate scenarios: *ie.* for 2030 and 2070, for two global climatic models, CSIRO-MK3.0 and Miroc-h, and two greenhouse gas emissions scenarios, A1B and A2 (4 future climate datasets for each year).

### CLIMEX

CLIMEX is a dynamic bioclimatic niche model that integrates modelled weekly responses of a population to climate in order to create a series of climatic suitability indices that can be mapped or graphed [28], [29]. CLIMEX calculates an annual Growth Index (GI_A_) to describe the potential for population growth as a function of weekly soil moisture and temperature during favourable conditions (Eq. 1).
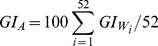
(1)where GI_W_ is the weekly Growth Index, composed of the weekly Temperature Index multiplied by the weekly Moisture Index. The Temperature Index and the Moisture Index are specified with a functional form that accords with the Law of Tolerance [38]–[40] and are combined in a multiplicative manner that accords with the Law of the Minimum [41]. It uses up to eight stress indices (cold, wet, hot, dry, cold-wet, cold-dry, hot-wet and hot-dry; respectively *CS, WS, HS, DS, CWX, CDX, HWX, HDX*) to estimate the ability of the population to survive unfavourable conditions (Eqs. 2,3).



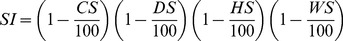
(2)

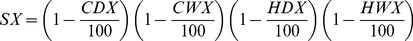
(3)


The Ecoclimatic Index (EI) summarises the balance between the opportunity for the species to grow during the favourable season(s) and the requirement to survive inclement season(s), and scales from 0 (unsuitable) to 100 (optimal) (Eq. 4).
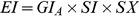
(4)


CLIMEX models are best informed by fitting the stresses to the boundary of the known range of the species, using knowledge, when available, about its biology or ecology to inform thresholds and rate parameters. The temperature and soil moisture weekly growth indices (*GI_w_*) were informed mostly by knowledge of the species biology and sometimes by using phenological observations and matched climates to infer growth limits and optima.

In our model, we tested the assumption that the presence of *C. imicola* in arid areas was due to irrigation maintaining suitable microclimate. The area under irrigation was identified using the data from Siebert *et al.* [42] describing presence of irrigation practices. In these areas we used CLIMEX irrigation scenario applying 3.5 mm per day as a “top-up”, *ie*. during dry season, irrigation increases the rainfall equivalent to this limit [29].

### Parameter-fitting, model validation and projection

Rainfall and temperature are known to drive activity, survival and seasonality of *Culicoides* [6], [43]. Initial values for the Growth and Stress parameters for the *C. imicola* CLIMEX model were informed by the following ecological traits. The species is present in warm and equatorial climatic conditions (Figure 1), but in regions where *C. imicola* is the main vector species for BT, it can persist over winter where daily maximum temperatures are greater than 12.5°C [44]–[46], and in Italy the probability of presence was close to zero where annual average of minimum daily temperatures fell below 9°C [47]. In laboratory studies Paweska *et al.* [9] noted that the minimum temperature for replication of bluetongue virus serotype 1 (BTV-1) in *C. imicola* was between 10°C and 15°C. In the Mediterranean region, the probability of *C. imicola* presence was greatest when minimum daily temperatures were greater than 16°C and increased with increasing relative humidity from 40% to its maximum at 60% [47]. In Morocco and Iberia [18], and in South Africa [22], minimum Normalized Difference Vegetation Index (NDVI), a variable correlated with soil moisture, is positively correlated with *C. imicola* abundance.

The natural rainfall model was fitted initially to *C. imicola*'s distribution only in the training zone until all occurrences receiving adequate rainfall to support a minimal amount of grass growth were modelled as being at least barely climatically suitable (minimising the climatically suitable area using biologically reasonable parameter values). The rationale for considering grass growth is that it is necessary to support the vertebrate hosts of *C. imicola*.

#### Cold Stress

We fitted a cold stress function to constrain survival to the coldest areas where *C. imicola* had been reported in traps.

Model parameters and intermediate steps in model development, successively using *training*, *verification* and *validation* datasets (Figure 1), are detailed in Appendix S2 in File S1.

The “immature model”, fitted to the native range distribution data, was tested against the *verification* area (Figure 1), comparing the region where EI>0 with occurrence records in South Eastern Europe, the Middle East and Asia. Where necessary, we adjusted the model during the verification step which resulted in a second model named the “completed model”. This step allowed us to make adjustments for any enemy release effects [48].

Each model was validated by calculating its sensitivity score: the proportion of records falling in locations modelled as being climatically suitable (EI>0). In addition, the degree of departure from random prediction was tested with the exact one-tailed binomial statistic [32] which measures the statistical significance of sensitivity scores given the modelled prevalence, *ie.* the proportion of the model universe that is climatically suitable. A useful model should have a high sensitivity score reflecting that recorded locations are modelled as being climatically suitable, and a significant binomial statistic score ensuring the model projection does not cover an excessive proportion of the study area. The completed model was then run using the global dataset under historical climate and future climate scenarios.

## Results

**Figure 2:**
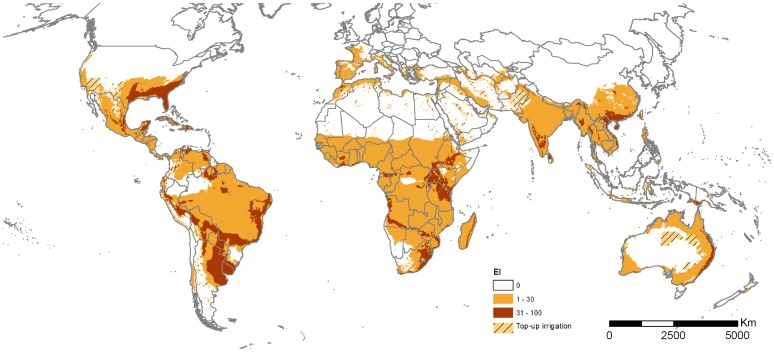
Global climate suitability for *Culicoides imicola* under historical conditions as represented by the CLIMEX Ecoclimatic Index (EI) for the completed model. Cross-hatching indicates suitable areas when irrigation is integrated in the model (Appendix S2 in File S1). Highly suitable areas in dark.

The geographical range of *C. imicola* appears primarily limited by cold stress and dry stress, and to a lesser extent wet stress. Its modelled potential distribution accords well with its known distribution in Africa, the Middle East and southern Europe (Figures 2, and SI2, SI3 and SI4 in File S1). The modelled climate suitability in France and continental Asia encompasses the reported distribution, but also extends well beyond the reported distribution. The model indicates large areas in the Americas and South-East Asia as being climatically suitable.

The probable role of irrigation in extending the potential range of *C. imicola* into xeric regions is indicated by how well the composite risk map improved the model sensitivity score without affecting the model specificity significantly (Table SI2, Figures SI2 and SI3 in Appendix S2 in File S1). For the three datasets, the completed model explained the presence of *C. imicola* in 833 grid cells at 10′ resolution and the addition of irrigation component explained an additional 38 records in arid areas (Appendix S2 in File S1). Only 8 occurrence records were not fitted in the composite irrigation model (Appendix S2, Figure SI4b in File S1).

**Figure 3:**
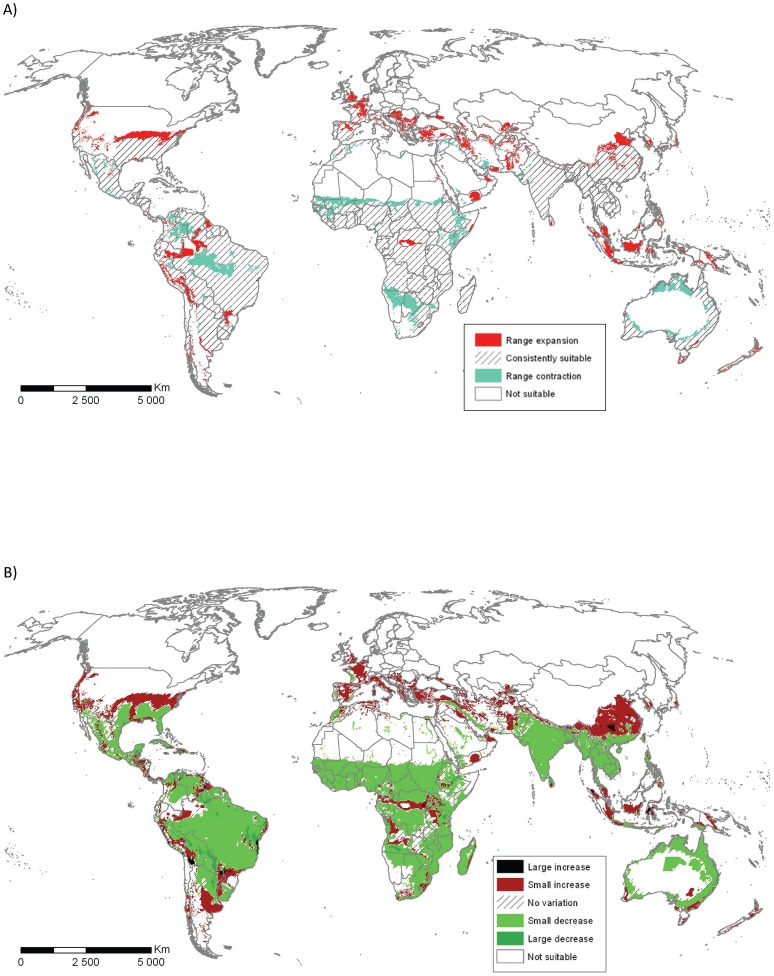
Forecast variation of ecoclimatic suitability between historical climate (current EI) and 2070 projected climate (future EI) from A2 climate scenarios using CSIRO-MK3.0 climate models. a) qualitative variation highlighted the expansion (current EI = 0, future EI>0) and the contraction (current EI>0, future EI = 0) of suitable areas for 2070 projection. b) quantitative variation illustrated climatic suitability increases or decreases. Small and large EI variations represented respectively differences in the range 3–30 and 31–100.

Under the natural rainfall future climate scenario, *C. imicola*'s projected potential distribution for climate scenario A2, generated by CSIRO-MK3.0 model, projected in 2070 (Figure 3) expands into areas that are presently too cold for persistence, and contracts from areas that are set to become more xeric. Similar changes in ecoclimatic suitability patterns were observed with other climate projections (*ie.* 2030 projections, A1B climatic scenario or Miroc-h climate model, see Figure SI5 and SI6 in Appendix S3, in File S1). In the scenarios considered, most of the potential range expansion due to a warming climate occurs in the Northern Hemisphere. In the Southern Hemisphere, where terrestrial habitat is limited to lower latitudes, most of the potential range expansion under the climate change scenario is into higher altitude areas. A similar response pattern was observed in equatorial regions (Figure 3).

## Discussion

There appears to be substantial opportunity for range expansion of *C. imicola* into the Americas and Australasia should it be translocated to these regions; the climate appears moderate to highly suitable over large areas, and domestic ruminant and equid hosts abound. Should *C. imicola* spread southwards into the Indonesian archipelago or Papua New Guinea, it is entirely feasible that it could spread to Australia [49], potentially increasing the risk of transmission of BT and AHS in Australia, and possibly the northern parts of New Zealand with a milder climate. In Australia, *C. imicola* would be adding to an extant community of competent vectors so the extent of the added risk is unknown.

The use of the irrigation information from Siebert *et al.* [42] allowed us to capture the effect of crop irrigation in supporting populations of *C. imicola* in xeric regions, without creating a risk map that indicated that vast areas of desert were climatically suited. The few location records in Figure 2 that fall in apparently climatically unsuitable locations are most likely the result of populations that are able to persist by virtue of small livestock watering points not captured by the irrigation dataset of Siebert *et al.* [42], perhaps signalling a scaling limitation regarding the use of this data. Agencies with biosecurity responsibilities in xeric regions may therefore wish to consider the irrigation climatic risk scenario independently of the irrigation locations indicated in Siebert *et al.* [42]. This risk map could indicate climate suitability for *C. imicola* in areas where soil moisture availability is not well modelled using natural rainfall and crop irrigation is not captured in the dataset of Siebert *et al.* [42].

The map of ecoclimatic suitability (Figure 2) approximates the fundamental niche of *C. imicola* (potential distribution negating limiting factors). As is commonly observed, the modelled potential distribution is wider than the observed distribution and the difference has several assumed origins:

1) Biotic factors, not included in our model, and especially presence and abundance of hosts which are important to maintain a blood feeding species such as *C. imicola*. Considering abundances of wild and domestic hosts is particularly important when modelling *C. imicola*'s abundance [16].
2) Incomplete sampling: apart from Europe, surveys have been implemented in a limited number of countries such as, for example, South Africa and Kenya, whereas other countries were under sampled (see point densities by countries in Figure 1 and Table SI1 in Appendix S1 in File S1). Further capture and identification of *Culicoides* species, especially in areas foreseen as highly suitable such as India and China, could improve our understanding of the ecoclimatic requirements of the species.
3) An ongoing (dynamic) process of colonization. Indeed, there have been no reports of *C. imicola* captured in the South West of France despite a dense and regularly surveyed trap network [36] (Figure SI4b in File S1); despite this area being modelled as suitable, and lies close to regions where some populations have been reported (South East of France, Venail *et al.* [36]; North West of Spain, Goldarazena *et al.* [50]). The colonization process is dependent on dispersal abilities, size of the source population, meteorological conditions and the presence of natural barriers. Venail *et al.* [36] discussed the confined distribution of *C. imicola* in South East France by physical altitudinal barriers.
4) Geographically isolated landmasses such as America and Australia with territories where ecoclimatic conditions are suitable and therefore insect establishment may be possible (however note that other processes, not considered here, such as competition and parasitism for example, could prevent for species establishment). Identification of such territories is relevant for biosecurity purposes: even if intercontinental dispersal of the vector is unlikely, importation of hitchhiking insects along with trade has been reported previously for other insects (*eg*. Caton *et al.*, [51]) and is well known for Aedine mosquitoes. Besides, the introduction of exotic serotypes in Northern Europe in 2008 and the novel emergence of Schmallenberg virus in the same area highlight our misunderstanding of the possible entry routes.

We found that *C. imicola* shares temperature requirements for development and survival with one Australasian *Culicoides*: optimal temperatures for survival of larva of *C. brevitarsis* range between 20 and 35.5°C (unfortunately temperatures lower than 20°C were not tested) and temperatures greater or equal 38°C are lethal [52]. Murray and Nix [53] found that adult activity of *C. brevitarsis* started at 12°C. The modelled range for survival of *C. imicola* in our study is 12–36°C (Figure SI1 in File S1). No information is available on moisture requirements for *C. brevitarsis* but the known distribution of *C. brevitarsis* [54] is similar to the projected potential distribution of *C. imicola* in Australia (Figure 2). Furthermore, other evidence relates the two species: phylogenetic analyses of different *Culicoides* species show that *C. brevitarsis* sampled from Japan [55] are closely related to *C. imicola* from South Africa [56], from France and Israel [57], [58]. Like *C. imicola*, *C. brevitarsis* is also competent for bluetongue virus [59]. Some authors have suggested a common *Culicoides* ancestral species for *C. brevitarsis* and *C. imicola* [35] but further study would be required to test this hypothesis. *Culicoides imicola* is the main vector of AHSV but we think that testing the competency of *C. brevitarsis* for AHSV would be informative for assessing biosecurity risks.

Under the future climate scenario, parts of Namibia, Botswana, Kenya, Somalia and Ethiopia could become unsuitable for *C. imicola* in the future, whereas the model projects a Northern expansion of suitable climate especially in Europe and China (Figure 3). However, presence of the vector is only one of the required conditions for vector-borne diseases to spread; appropriate hosts and presence of the pathogen are also required along with climatic conditions enabling vector activity, in particular vector-host contact, and pathogen replication. A complete risk assessment should also consider host densities and their relationship with vector densities [60], [61] and account for transmission routes from infected areas [49], [62].

Our study enables us to indirectly infer current and future risks of bluetongue and African horse sickness, diseases both transmitted by *C. imicola*, and thereby provides a framework for updating management and biosecurity strategies to target disease epizootics.

## Supporting Information

File S1 (PDF)
