# Correction: Worldwide Niche and Future Potential Distribution of *Culicoides imicola*, a Major Vector of Bluetongue and African Horse Sickness Viruses

**DOI:** 10.1371/journal.pone.0119323

**Published:** 2015-03-23

**Authors:** The PLOS ONE Staff

There are errors in the Funding section. Please see the complete, correct Funding statement here: This work was jointly funded by the Project “Climate Change and Impact Research: the Mediterranean Environment (CIRCE),” European Commission’s 6th Framework Programme and also by HarvestChoice project with support of the Commonwealth Scientific and Industrial Research Organization (CSIRO), the University of Minnesota and the Bill and Melinda Gates Foundation. This study was partially funded by EU grant FP7–261504 EDENext and is catalogued by the EDENext Steering Committee as EDENext199 (http://www.edenext.eu). The contents of this publication are the sole responsibility of the authors and don’t necessarily reflect the views of the European Commission. H. Guis, T. Balenghien and C. Garros were funded by the Ministère de l’agriculture, de l’agroalimentaire et de la forêt. The funders had no role in study design, data collection and analysis, decision to publish, or preparation of the manuscript.

There is an error in the first sentence of the Acknowledgements section. The correct sentence is: We thank J.C. Delécolle and T. Baldet for providing unpublished data and valuable comments[1].
