# Supplementary material for: Worldwide Niche and Future Potential Distribution of Culicoides imicola, a Major Vector of Bluetongue and African Horse Sickness Viruses
Source: PLoS One. 2014 Nov 12;9(11):e112491. doi: 10.1371/journal.pone.0112491 (PMC4229218; doi:10.1371/journal.pone.0112491)
Supplement: File S1 — (PDF) [file pone.0112491.s001.pdf]

Worldwide niche and future potential distribution of *Culicoides imicola*, a major vector of bluetongue and African horse sickness viruses: SUPPORTING INFORMATION

Sylvain Guichard[1,2], Hélène Guis[3,4], Annelise Tran[1,5], Claire Garros[3,4], Thomas Balenghien[3,4], Darren J. Kriticos[2,6,#]

[1] *Cirad, UR AGIRs, F-34398 Montpellier, France*

[2] *InSTePP, University of Minnesota, St. Paul, MN, USA*

[3] *Cirad, UMR CMAEE, F-34398 Montpellier, France*

[4] *INRA, UMR1309 CMAEE, F-34398 Montpellier, France*

[5] *Cirad, UMR15 TETIS, F-34398 Montpellier, France*

[6] *CSIRO Agricultural Productivity Flagship and Biosecurity Flagship, GPO Box 1700 Canberra, ACT 2601*

[#] Corresponding author, darren.kriticos@csiro.au

Appendix S1: Database of insect distribution and references

Appendix S2: Model development

Appendix S3: Climate change scenario projections

Appendix S1: Database of insect distribution and references

A geo-database of 1 381 occurrences of *Culicoides imicola*, covering 1959 to 2010, South Africa to North Europe and East Africa to China was compiled from 74 references (Table SI1). *Culicoides imicola* is part of an Imicola complex along with 10 other formally described species [1–3]. *Culicoides imicola* was firstly described in Kenya (Kieffer, 1913) and several names were eventually raised to synonymy [4]: *C. pallidipennis* (Carter et al., 1920), *C. iraqensis* (Khalaf, 1957) , *C. minutus* (Das Gupta, 1962) and *C. pseudoturgidus* (Sen and Das Gupta, 1959). The *C. imicola* taxon is well described [5,6]. In the present study, occurrences published under one of these synonymic names were subsequently named *C. imicola*.

Geographical coordinates were derived from published coordinates, locality coordinates (654 data), or maps (727 data). In the latter case, maps were imported as digital scans in ArcGIS software and georeferenced. We subsequently up-scaled the data at 10' granularity using CliMond's spatial grid [7], resulting in a geo-database of 879 distinct cells where the insect has been recorded. When several occurrences were recorded in a single 10’ cell, one record only was kept ensuring consistent resolution with climate data and removing duplicate records published several times.

Table SI1: Reported presence of *Culicoides imicola* extracted from publications on a country basis.

Country	Number of occurrences	Reference
Algeria	12	[Delécolle, unpublished data,2,3]
Angola	3	[10]
Bahrain	1	[11]
Botswana	2	[12,13]
Cape Verde	1	[14]
Chad	1	[15]
China	1	[16]
Congo	3	[17–19]
Egypt	4	[20,15]
Ethiopia	1	[15]
France	25	[8,21]
France (Reunion Island)	17	[22]
Gambia	4	[18,15]
Ghana	1	[15]
Greece	40	[23,24,8,25]
Burkina Faso	1	[26]
India	4	[27]

Country	Number of occurrences	Reference
Iran	1	[28]
Iraq	1	[12]
Israel	45	[29–32,23,15,24]
Italy	510	[33,34,8,35,36]
Kenya	54	[37,38,15,39,40]
Laos	2	[41,42]
Lesotho	1	[43]
Madagascar	2	[44,45]
Malawi	2	[12]
Mauritius	3	[46,15]
Morocco	29	[47,48,8]
Namibia	1	[12]
Nigeria	5	[18,15,49,50]
Oman	4	[11]
Portugal	85	[51,52,23,24,8,53]
Saudi Arabia	15	[54,11,55]
Senegal	6	[26]
Sicily	8	[8]
South Africa	120	[56,23,57–59,12,60,24,45,61,62,43]
Spain	283	[63–66,8,67–69,53,70]
Sudan	4	[71,72]
Sultanate of Oman	6	[73][73,74]
Swaziland	1	[12]
Switzerland	1	[75]
Tanzania	2	[39]
Thailand	13	[42]
Tunisia	10	[76,77,8]
Turkey	22	[78,8]
Uganda	2	[39]
United Arab Emirates	2	[11,8]
Vietnam	1	[42]
Yemen	4	[11]
Zimbabwe	15	[79,15,80]

Appendix S2: Model development

Parameters of temperature, soil moisture and cold stress indexes (Figure SI1) were adjusted from initial values to match modelled survival (*ie.* positive EI) with reported occurrences.

The model was firstly developed only on training dataset, and then tested on verification dataset and finally tested using the validation dataset (Figure 1). At each step the goodness of fit and sensitivity were evaluated following Anderson *et al*. [81] and Webber *et al*. [82] (Table SI2).

Training dataset

Fitted on *C. imicola*'s distribution, the Ecoclimatic Index (EI) of the "immature" CLIMEX model matched 178 occurrences of the 187 occurrences for the training dataset. The unfitted occurrences located in South Africa, Sudan and Ethiopia (respectively 6, 2 and 1 data) were located in arid areas, *ie.* aridity index below 0.2 [83,84], apparently under irrigation (Figure SI2a). These outliers fall in areas that according to Google Earth have irrigation activities (circular green structures or green patchwork against a brown background matrix) in areas with extremely little rainfall. We added an irrigation component based on the presence of irrigation equipments [85,86] to reflect impact of human activity of irrigation on microclimate (Figure SI2a). With an irrigation component the model fitted the entire 187 occurrences of the training dataset (Figure SI2b).

Culicoides imicola is widespread in the training zone (covering the native range) and the model sensitivity was significant given a high prevalence value (Table SI2). To test it, the immature model was then projected on the verification area.

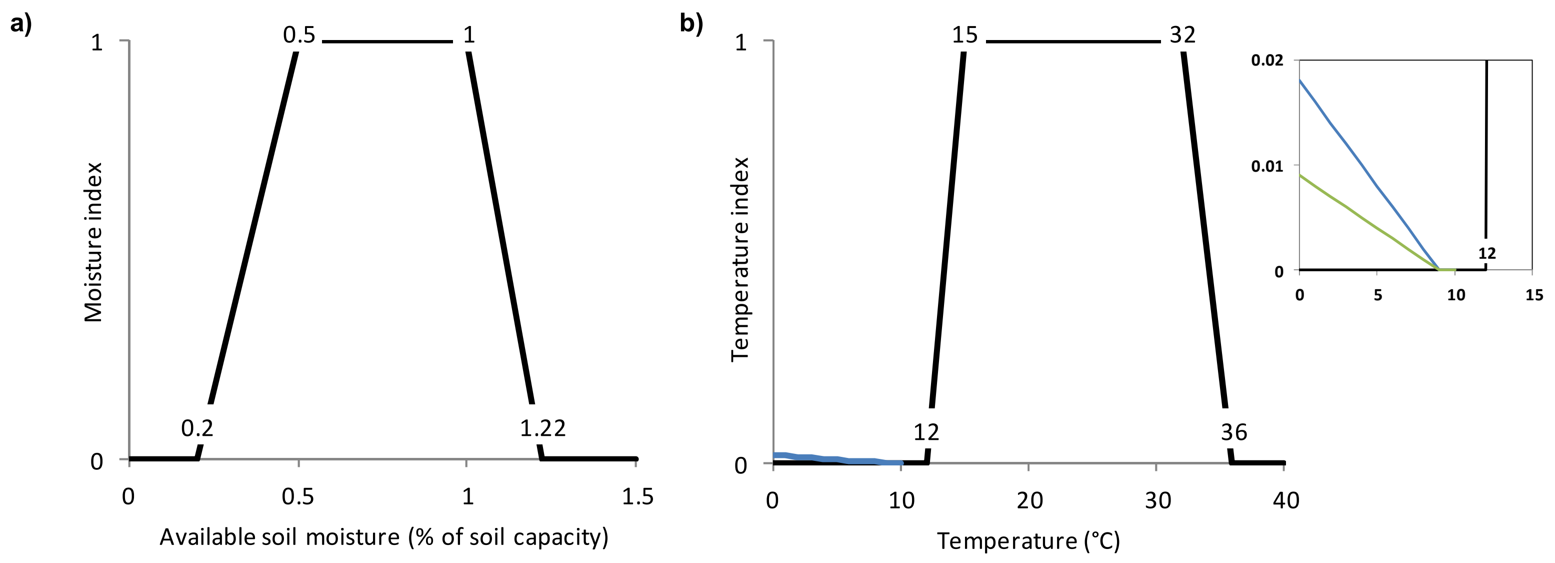

Figure SI1: CLIMEX parameters. a) soil moisture index, b) temperature index, insert: cold stress for the immature model (blue line) and for the completed model (green line)

Table SI2: Goodness of fit and sensitivity score for the different models using independent datasets. †: areas and occurrences of *Culicoides imicola* are expressed at 10' by 10' granularity. **: p-value<0.01.

Dataset	Extent (occurrences)†	Model	Suitable area†	True positive†	False negative†	Modelled prevalence, *ie.* chance expectation	Sensitivity score [confidence interval]
Training	60958 (187)	Immature	53867	178	9	0.88	0.95** [0.910 ; 0.977]
		Immature + irrigation	54532	187	0	0.89	1.00** [0.980; 1.000]
Verification	74897 (131)	Definitive	25895	105	26	0.35	0.80** [0.722 ; 0.866]
		Definitive + irrigation	30572	129	2	0.41	0.98** [0.945 ; 0.998]
Validation	21217 (561)	Definitive	5950	550	11	0.28	0.98** [0.965 ; 0.990]
		Definitive + irrigation	6713	555	6	0.32	0.99** [0.976 ; 0.996]

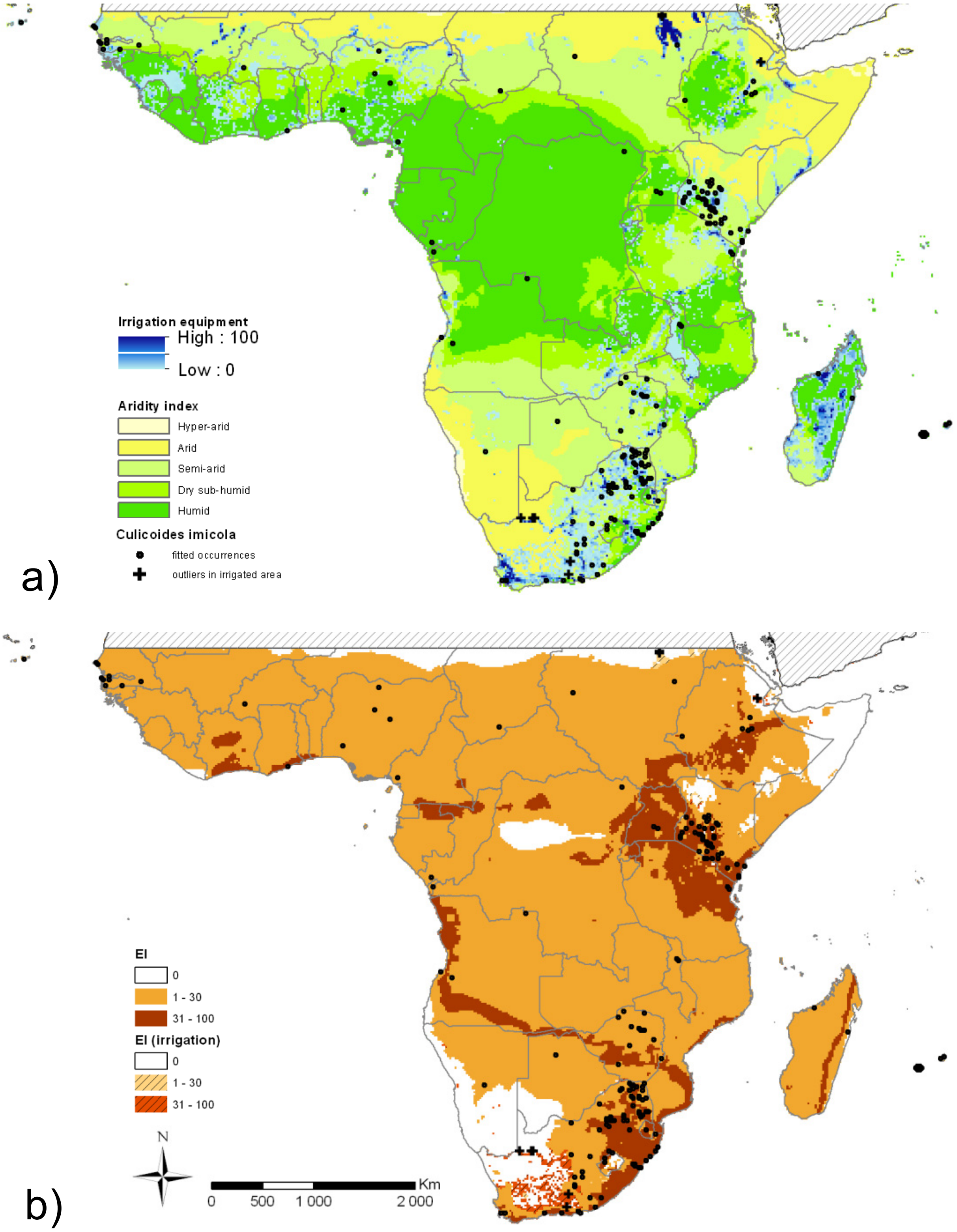

Figure SI2: Adjustment of the "immature" model in sub-Saharan Africa. a) Distribution of *Culicoides imicola*' occurrences (dots and crosses representing 187 cells at 10' resolution), presence of irrigation equipment [85,86] and aridity index [83,84] for the training zone. b) Ecoclimatic Index (EI) ranging from unsuitable (EI=0) to optimal (EI between 31 and 100) of the immature model only or with irrigation component (hatched).

Verification dataset

The projection of the immature model is shown in Figure SI3a with 5 unfitted locations highlighted with red crosses versus 126 locations correctly modelled as suitable (black symbols). All the 19 grid cells from the verification dataset located in equatorial climate (points located in China, Thailand, India and Vietnam; Figure 1) were modelled as suitable. For the 3 unfitted points located in Greece, the survival was limited by cold stress and subsequently we adjusted it by slightly reducing the impact of cold stress (parameter change in the insert of Figure SI1, completed model in Figure SI3b). The other 2 unfitted occurrences were located in the Arabian Peninsula, areas experiencing an arid climate (Figure SI3a) but where there was no evidence of irrigation to explain the presence of the insect at these locations whereas the irrigation scenario correctly reclassified 24 outliers from Egypt to the Sultanate of Oman (black crosses in Figure SI3).

The goodness of fit of the completed model is high given a small prevalence value of the verification dataset (Table SI2).

Validation dataset

The strength of our model was confirmed after projection in the validation area where the sensitivity score was also close to 1 given a prevalence value around 0.3 (Table SI2). Only 6 locations remained unfitted (highlighted by red crosses in Figure SI4) out of 561 occurrences in total. One unfitted occurrence was located in Algeria in a 10' grid cell where there was no evidence of irrigation (Figure SI4a). Four other unfitted points were located in Spain and Italy at the fringe of the suitable area which could be explained by local dispersion of the insect. The last unfitted occurrence represented the capture of one *C. imicola* in a single trap in Switzerland [75], probably the result of a rare event of long distance dispersal. The figure SI4b also highlights 10’ cells in which only absences of *C. imicola* were reported [47,48,75,63,51,52,87,76,34,65,66,69,88,89,70,9,21] and illustrates the sampling effort in Europe. The European and North African absence data must be interpreted in the context of an active biological invasion. This means that some of the apparent absences may be suitable for *C.*

imicola but have not yet trapped this species due to an invasion lag or the existence of an unsuitable non-climatic habitat factor [90]. Without additional information, it is impossible to distinguish such locations from those that are climatically unsuitable. The difference between the modelled potential distribution and observed distribution is discussed in the main manuscript.

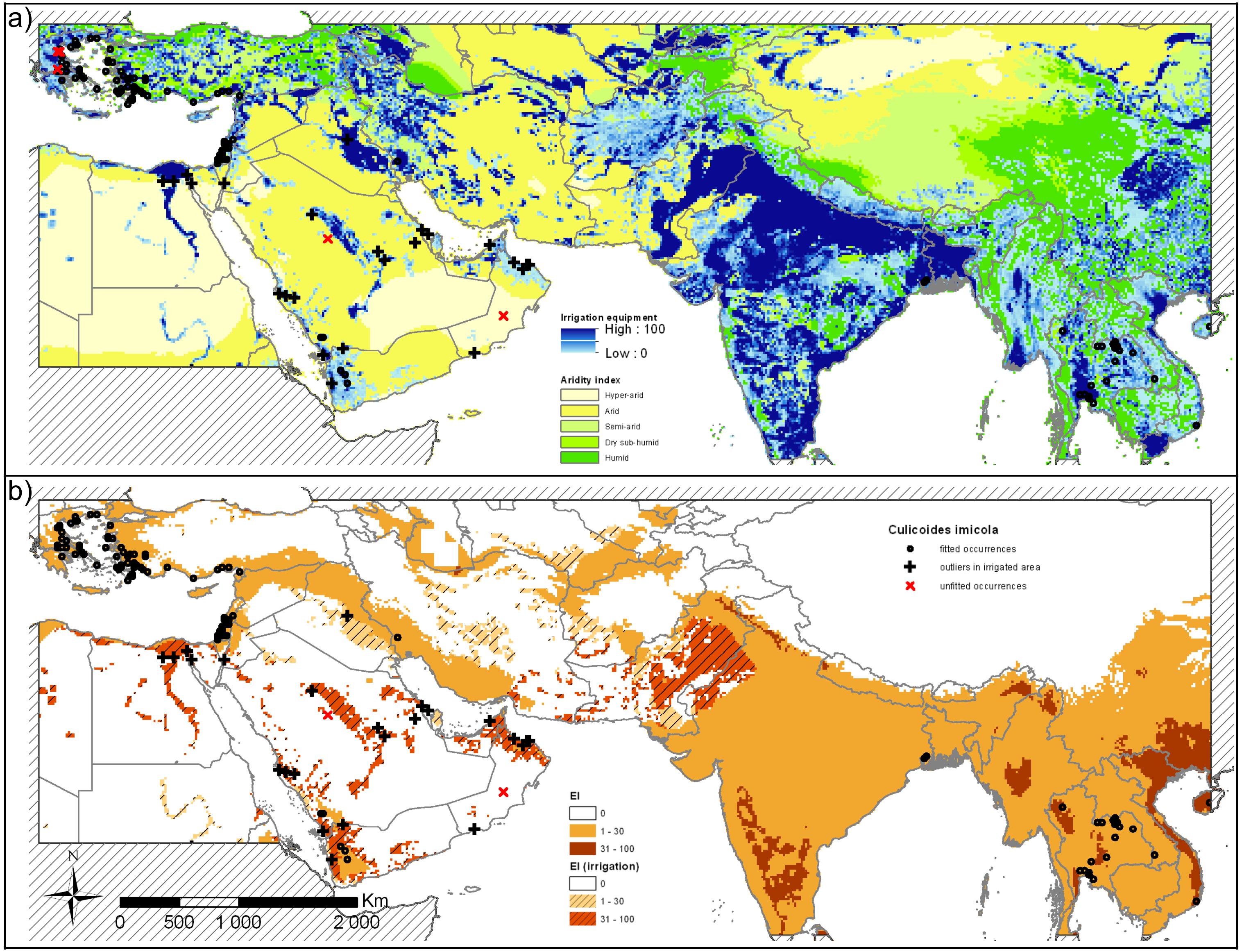

Figure SI3: Verification step in the modelling process. Distribution of *Culicoides imicola'* occurrences (10'-grid cells represented by dots and crosses, 131 occurrences in total). a) representation of fitted occurrences for the immature model only (black dots, n=105) and with an irrigation component (black dots and black crosses, n=126) for the verification zone. Unfitted occurrences are represented by red crosses (n=5 with 3 occurrences in Greece). Background highlighted irrigation equipment and aridity index. b) Ecoclimatic index (EI) ranging from unsuitable (EI=0) to optimal (EI between 31 and 100) of the completed model only or with irrigation component (hatched). Tuning the cold stress index (Figure SI1b) allowed the three previously unfitted occurrences in Greece (Figure SI3a) with the so-called completed model to be fitted correctly.

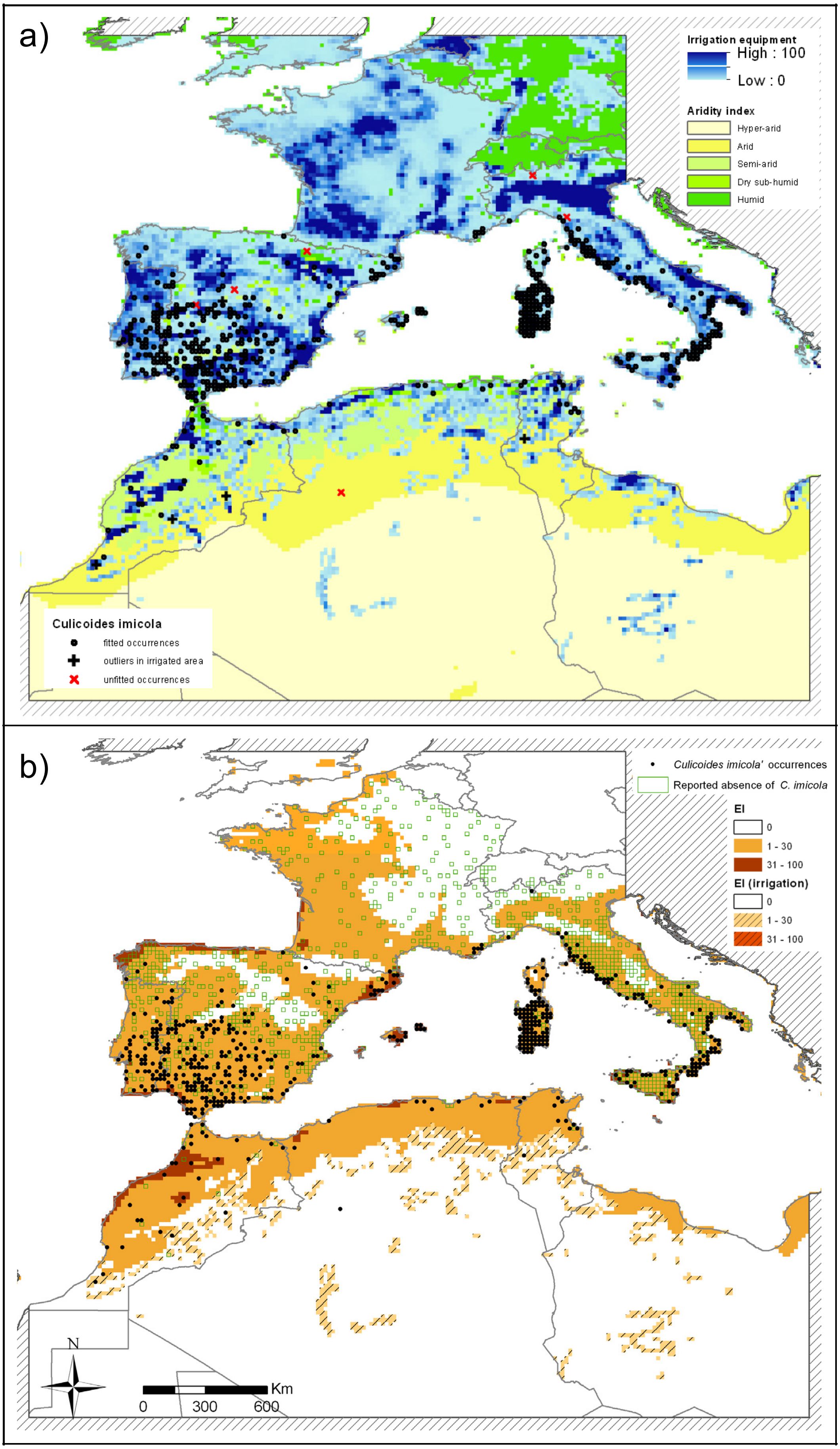

Figure SI4: Climate suitability for *Culicoides imicola* under historical conditions as represented by the CLIMEX Ecoclimatic Index for the completed model across the validation extent. a) Black crosses and dots indicated a suitable Eco-climatic Index at locations where *C. imicola* has been trapped for CLIMEX models respectively with irrigation component or without, and red crosses indicated positive *C. imicola* trap catches with a modelled unsuitable climate. Background highlighted irrigation equipment and aridity index. b) Ecoclimatic index (EI) ranging from unsuitable (EI=0) to optimal (EI between 31 and 100) of the completed model only or with irrigation component (hatched). Black dots represent trap catches of *C. imicola* and green squares the reported absence of *C. imicola*

Appendix S3: Climate change scenario projections

**2030<br>A2<br>CS**	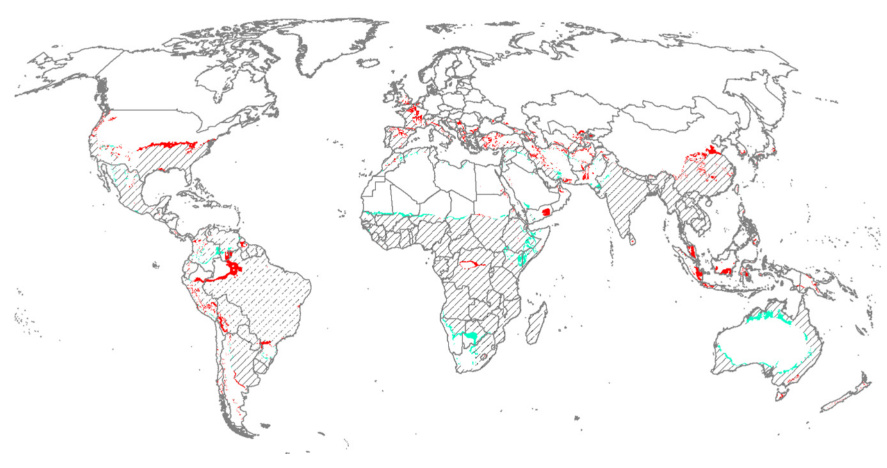
**2030<br>A2<br>MR**	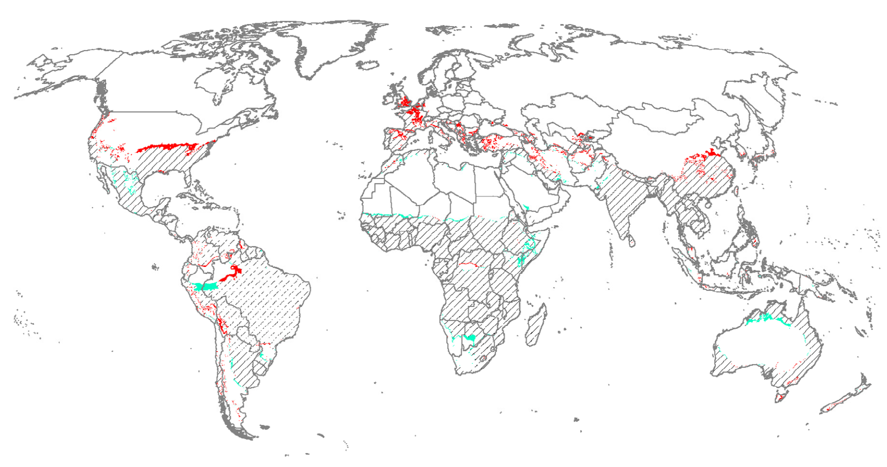
**2030<br>A1B<br>CS**	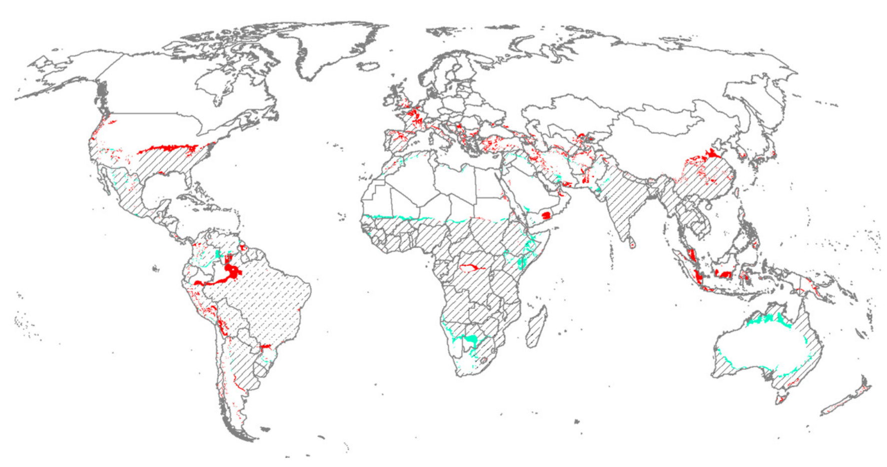
**2030<br>A1B<br>MR**	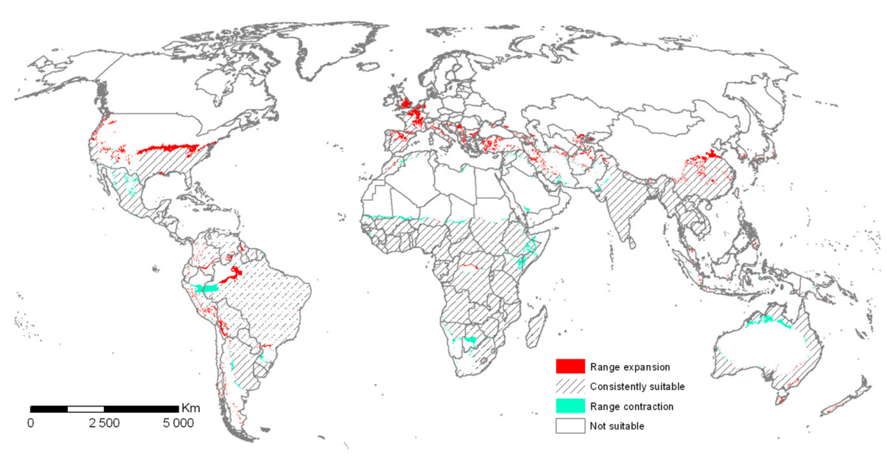

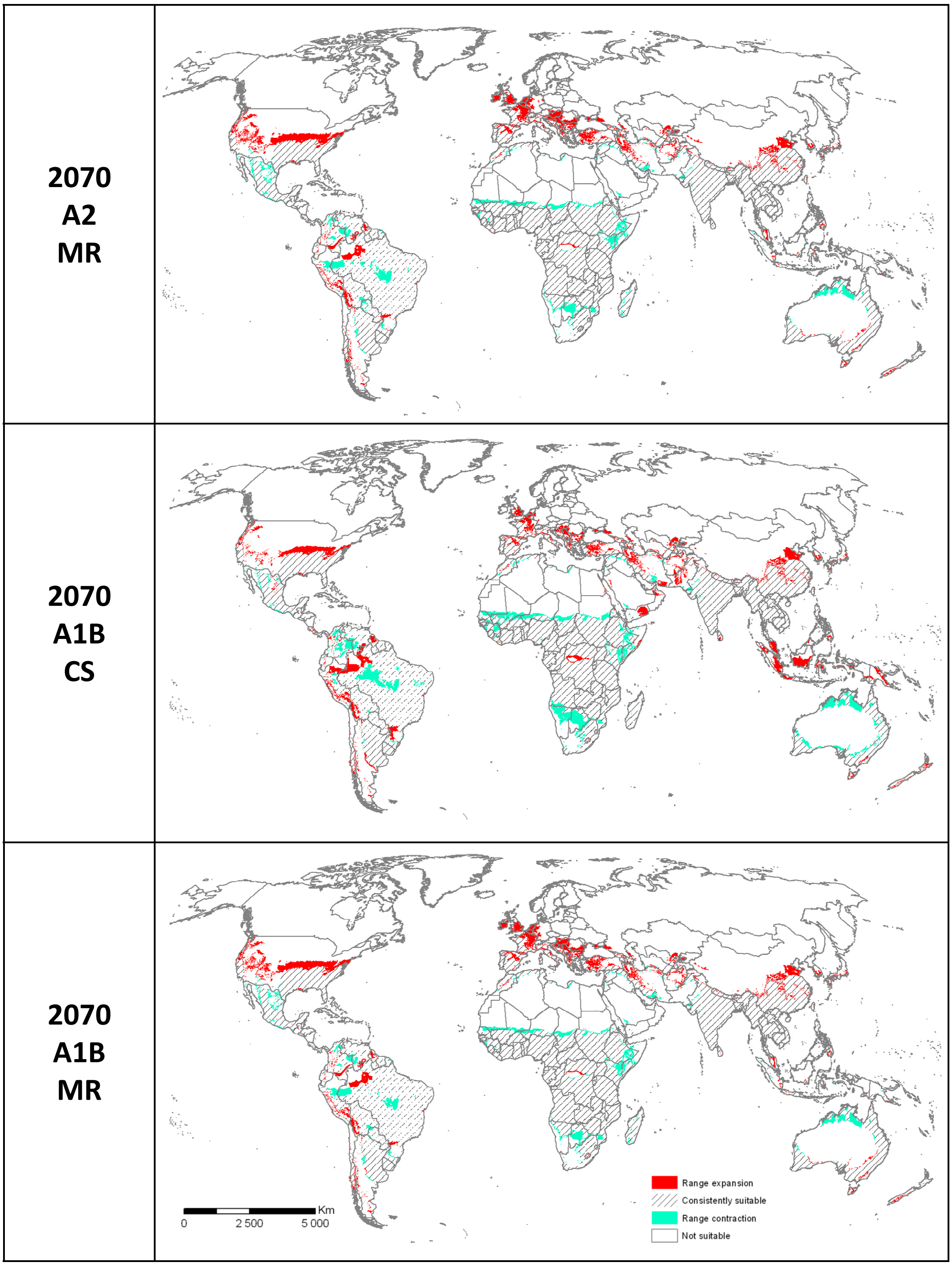

Figure SI5: Forecast variation of ecoclimatic suitability between historical climate (current EI) and 2030 or 2070 projected climate (future EI) from A1B or A2 climate scenarios using CSIRO-MK3.0 or Miroc-h climate models (respectively abbreviated as CS and MR). Qualitative variation highlighted the expansion (current EI=0, future EI>0) and the contraction (current EI>0, future EI=0) of suitable areas for the projection. The forecast of qualitative variation for 2070/A2/CS future climate was represented in Figure 3a.

2030
A2
CS

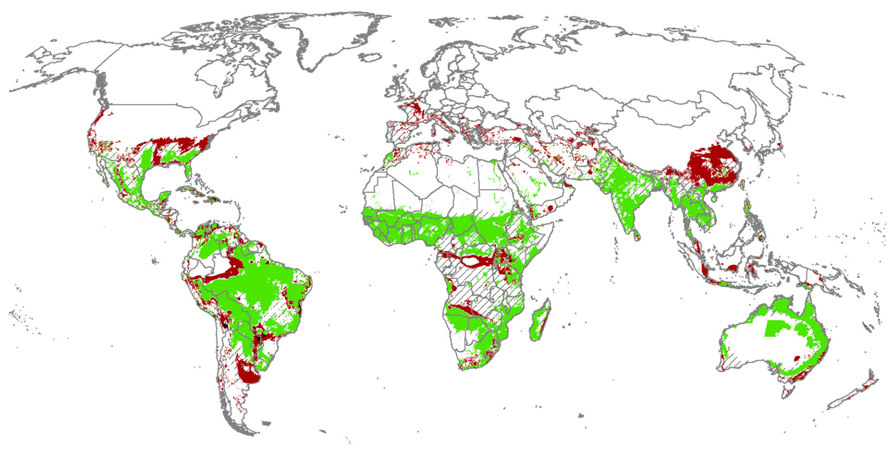

2030
A2
MR

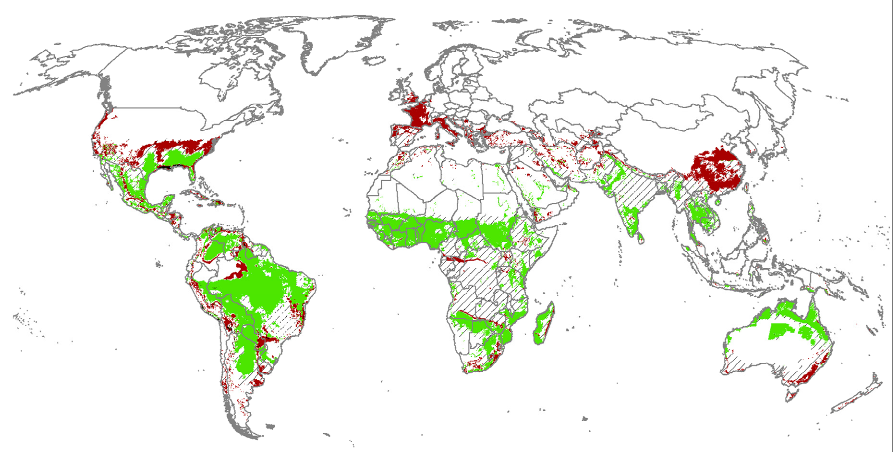

2030
A1B
CS

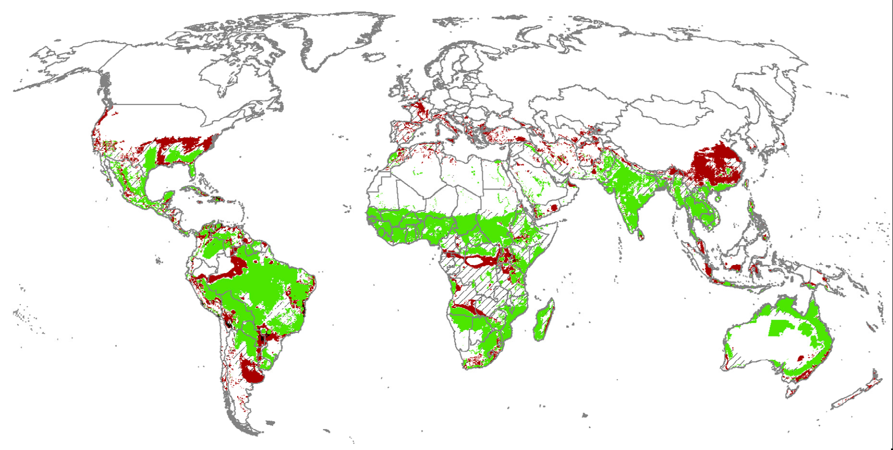

2030
A1B
MR

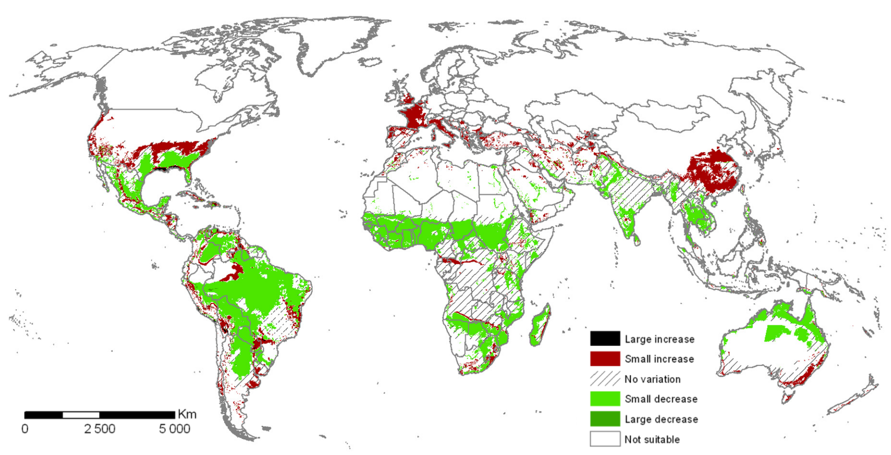

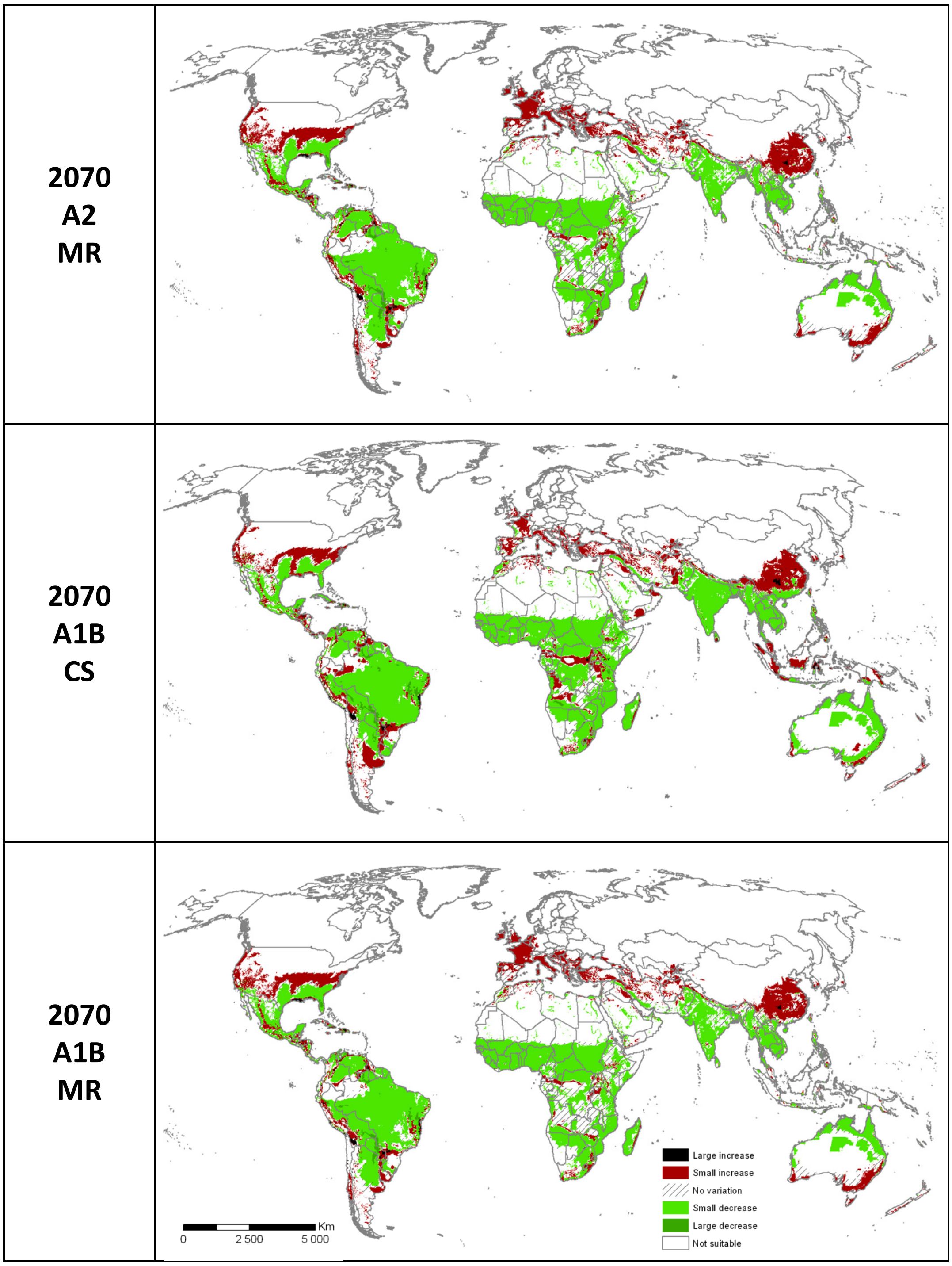

Figure SI6: Forecast variation of ecoclimatic suitability between historical climate (current EI) and 2030 or 2070 projected climate (future EI) from A1B or A2 climate scenarios using CSIRO-MK3.0 or Miroc-h climate models (respectively abbreviated as CS and MR). Quantitative variation illustrated climatic suitability increases or decreases. Small and large EI variations represented respectively differences in the range 3-30 and 31-100. The forecast of quantitative variation for 2070/A2/CS future climate is presented in Figure 3b.

References for supporting information:

1. Bellis G, Dyce A, Gopurenko D, Yanase T, Garros C, et al. (2014) Revision of the *Culicoides (Avaritia)* Imicola complex Khamala & Kettle (Diptera: Ceratopogonidae) from the Australasian region. Zootaxa 3768: 401–427.

2. Meiswinkel R (2004) Adult characters defining and separating the Imicola and Orientalis species complexes of the subgenus *Avaritia* Fox, 1955 (*Culicoides*, Diptera: Ceratopogonidae). Veterinaria Italiana 40: 345–351.

3. Nevill H, Venter GJ, Meiswinkel R, Nevill EM (2007) Comparative descriptions of the pupae of five species of the *Culicoides imicola* complex (Diptera, Ceratopogonidae) from South Africa. Onderstepoort Journal of Veterinary Research 74: 97–114.

4. Borkent A (2014) World Species of Biting Midges (Diptera: Ceratopogonidae). http://www.inhs.illinois.edu/research/FLYTREE/Borkent.html.

5. Delécolle JC, De La Rocque, S (2002) Contribution à l'étude des *Culicoides* de Corse. Liste des espèces recensées en 2000/2001 et redescription du principal vecteur de la fièvre catarrhale ovine : *C. imicola* Kieffer, 1913 (Diptera, Ceratopogonidae). Bulletin de la Société entomologique de France 107: 371–379.

6. Meiswinkel R, Gomulski LM, Delécolle J-C, Goffredo M, Gasperi G (2004) The taxonomy of *Culicoides* vector complexes - unfinished business. Veterinaria Italiana 40: 151–159.

7. Kriticos DJ, Webber BL, Leriche A, Ota N, Macadam I, et al. (2012) CliMond: global high-resolution historical and future scenario climate surfaces for bioclimatic modelling. Methods in Ecology and Evolution 3: 53–64.

8. Nolan DV, Dallas JF, Pertney SB, Mordue (Luntz) AJ (2008) Incursion and range expansion in the bluetongue vector *Culicoides imicola* in the Mediterranean basin: a phylogeographic analysis. Medical and Veterinary Entomology 22: 340–351.

9. Szadziewski R (1984) Ceratopogonidae (Diptera) from Algeria. VI. *Culicoides* Latr. Polskie Pismo Entomologiczne 54: 163–182.

10. Kremer M (1972) *Culicoides* (Diptera : Ceratopogonidae) de la région éthiopienne et particulièrement d'Angola (IIe note) (espèces, nouvelles, redescription et chorologie). Publicações culturais da Companhia de Diamantes de Angola 84: 79–108.

11. Boorman J (1989) *Culicoides* (Diptera: Ceratopogonidae) of the Arabian Peninsula with notes on their medical and veterinary importance. Fauna of Saudi Arabia 10: 160–224.

12. Meiswinkel R (1989) Afrotropical *Culicoides*: a redescription of *C. (Avaritia) imicola* Kieffer, 1913 (Diptera: Ceratopogonidae) with description of the closely allied *C. (A.) bolitinos* sp. nov. reared from the dung of the African buffalo, blue wildebeest and cattle in South Africa. Onderstepoort Journal of Veterinary Research 56: 23–39.

13. Mushi EZ, Isa JFW, Chabo RG, Binta MG, Kapaata RW (1998) *Culicoides* (Diptera: Ceratopogonidae) associated with horses at Mogoditshane, Gaborone, Botswana. Veterinary Research Communications 22: 295–297.

14. Boorman J, Vanharten A (1992) Vectors of african horse sickness in the Cape Verde Islands. Veterinary Record 131: 56–56.

15. Glick JI (1990) *Culicoides* biting midges (Diptera: Ceratopogonidae) of Kenya. Journal of Medical Entomology 27: 85–195.

16. Yu Y (2005) Ceratopogonidae of China: Insecta, Diptera. Beijing: Military Medical Science Press (in Chinese).

17. Clastrier J (1960) Notes sur les Cératopogonidés. IX. Cératopogonidés de la République du Congo. Archives de l'Institut Pasteur d'Algérie 38: 79–105.

18. Clastrier J, Wirth WW (1961) Notes sur les Cératopogonidés. XIV. Cératopogonidés de la région éthiopienne. Archives de l'Institut Pasteur d'Algérie 39: 302–337.

19. Itoua A, Cornet M (1986) Les Ceratopogonidae (Diptera) du Mayombe congolais. 3. Revue taxonomique des espèces du genre *Culicoides* Latreille, 1809. Cahiers ORSTOM Entomologie Médicale et Parasitologie 24: 233–250.

20. Ghonaim MF, Fadl HH, Ibrahim AA, Ali. A (2001) An annotated checklist of Ceratopogonidae (Diptera) of Egypt. Oriental Insects 35: 247–258.

21. Venail R, Balenghien T, Guis H, Tran A, Setier-Rio M-L, et al. (2012) Assessing diversity and abundance of vector populations at a national scale: example of *Culicoides* surveillance in France after bluetongue virus emergence. In: Mehlhorn H, editor. Arthropods as vectors of emerging diseases. Parasitology Research Monographs. Springer Berlin Heidelberg, Vol. 3. pp. 77–102.

22. Desvars A (2005) Etude préliminaire des espèces de *Culicoides* (Diptera : Ceratopogonidae), vecteurs avérés ou potentiels d'arboviroses animales, à l'île de La Réunion. Université Montpellier I.

23. Dallas JF, Cruickshank RH, Linton Y-M, Nolan DV, Patakakis M, et al. (2003) Phylogenetic status and matrilineal structure of the biting midge, *Culicoides imicola*, in Portugal, Rhodes and Israel. Medical and Veterinary Entomology 17: 379–387.

24. Nolan DV, Dallas JF, Mordue (Luntz) AJ (2004) Molecular taxonomy and population structure of a *Culicoides* midge vector. Veterinaria Italiana 40: 352–359.

25. Patakakis MJ, Papazahariadou M, Wilson A, Mellor PS, Frydas S, et al. (2009) Distribution of *Culicoides* in Greece. Journal of Vector Ecology 34: 243–251.

26. Cornet M (1969) Les Culicoïdes (*Diptera Ceratopogonidae*) de l'Ouest africain (1ère note). Cahiers ORSTOM Série Entomologie Médicale et Parasitologie 7: 341–364.

27. Dyce AL, Wirth WW (1983) Reappraisal of some Indian *Culicoides* species in the subgenus *Avaritia* (Dipteria: Ceratopogonidae). International Journal of Entomology 25: 221–225.

28. Navai S, Mesghali A (1968) Ceratopogonidae (Diptera) of Iran - II. More records of *Culicoides* Latreille, 1809. Journal of Natural History 2: 241–246.

29. Braverman Y (1988) Preferred landing sites of *Culicoides* species (Diptera: Ceratopogonidae) on a horse in Israel and its relevance to summer seasonal recurrent dermatitis (sweet itch). Equine Veterinary Journal 20: 426–429.

30. Braverman Y, Boorman J, Kremer M, Delécolle JC (1976) Faunistic list of *Culicoides* (Diptera, Ceratopogonidae) from Israel. Cahiers ORSTOM Entomologie Médicale et Parasitologie 14: 179–185.

31. Braverman Y, Chechik F, Mullens BA (2001) The interaction between climatic factors and bluetongue outbreaks in Israel and the Eastern Mediterranean, and the feasability of establishing bluetongue-free zones. Israel Journal of Veterinary Medicine 56: 99–109.

32. Braverman Y, Linley JR (1993) Effect of light trap height on catch of *Culicoides* (Diptera: Ceratopogonidae) in Israel. Journal of Medical Entomology 30: 1060–3.

33. Calistri P, Goffredo M, Caporale V, Meiswinkel R (2003) The distribution of *Culicoides imicola* in Italy: Application and evaluation of current Mediterranean models based on climate. Journal of Veterinary Medicine Series B 50: 132–138.

34. Conte A, Gilbert M, Goffredo M (2009) Eight years of entomological surveillance in Italy show no evidence of *Culicoides imicola* geographical range expansion. Journal of Applied Ecology 46: 1332–1339.

35. Pili E, Ciuccè S, Culurgioni J, Figus V, Pinna G, et al. (2006) Distribution and abundance of Bluetongue vectors in Sardinia: Comparison of field data with prediction maps. Journal of Veterinary Medicine, Series B 53: 312–316.

36. Purse BV, Tatem AJ, Caracappa S, Rogers DJ, Mellor PS, et al. (2004) Modelling the distributions of *Culicoides* bluetongue virus vectors in Sicily in relation to satellite-derived climate variables. Medical and Veterinary Entomology 18: 90–101.

37. Davies FG (1978) Bluetongue studies with sentinel cattle in Kenya. Journal of Hygiene (Cambridge) 80: 197–204.

38. Davies FG, Walker AR, Ochieng P, Shaw T (1979) Arboviruses isolated from *Culicoides* midges in Kenya. Journal of Comparative Pathology 89: 587–595.

39. Khamala CPM (1971) Ecological distribution of East African *Culicoides* Latreille (Dipt., Ceratopogonidae) as shown by light-traps. Bulletin of Entomological Research 60: 549–557.

40. Walker AR (1977) Seasonal fluctuations of *Culicoides* species (Diptera: Ceratopogonidae) in Kenya. Bulletin of Entomological Research 67: 217–233.

41. Howarth FG (1985) Biosystematics of the *Culicoides* of Laos (Diptera: Ceratopogonidae). International Journal of Entomology 27: 1–96.

42. Wirth WW, Hubert AA (1989) The *Culicoides* of Southeast Asia (Diptera: Ceratopogonidae). Washington DC: Walter Reed Army Institute of Research.

43. Venter GJ, Nevill EM, Van Der Linde TC de K (1996) Geographical distribution and relative abundance of stock-associated *Culicoides* species (Diptera:Ceratopogonidae) in southern Africa, in relation to their potential as viral vectors. Onderstepoort Journal of Veterinary Research 63: 25–38.

44. De Meillon B (1961) The Madagascan Ceratopogonidae. Revista de Entomologia de Moçambique 4: 37–64.

45. Sebastiani F, Meiswinkel R, Gomulski LM, Guglielmino CR, Mellor PS, et al. (2001) Molecular differentiation of the Old World *Culicoides imicola* species complex (Diptera, Ceratopogonidae), inferred using random amplified polymorphic DNA markers. Molecular Ecology 10: 1773–1786.

46. Boorman J, Mellor PS (1992) *Culicoides* vectors of bluetongue and African horse sickness viruses in Mauritius. Medical and Veterinary Entomology 6: 306–306.

47. Bailly-Choumara H, Kremer M, Delécolle JA (1970) Deuxième contribution à l'étude des *Culicoides* du Maroc (Diptera, Ceratopogonidae). Cahiers ORSTOM Série Entomologie Médicale et Parasitologie 8: 383–391.

48. Baylis M, El Hasnaoui H, Bouayoune H, Touti J, Mellor PS (1997) The spatial and seasonal distribution of African horse sickness and its potential *Culicoides* vectors in Morocco. Medical and Veterinary Entomology 11: 203–212.

49. Herniman KA, Boorman JP, Taylor WP (1983) Bluetongue virus in a Nigerian dairy cattle herd. 1. Serological studies and correlation of virus activity to vector population. Journal of Hygiene (Cambridge) 90: 177–193.

50. Kitaoka S, Kaneko K, Shinonaga S (1984) Survey on blood-sucking *Culicoides* species in Ife, Nigeria. Journal of the Aichi Medical University Association 12: 454–458.

51. Capela R, Purse BV, Pena I, Wittman EJ, Margarita Y, et al. (2003) Spatial distribution of *Culicoides* species in Portugal in relation to the transmission of African horse sickness and bluetongue viruses. Medical and Veterinary Entomology 17: 165–77.

52. Capela R, Sousa C, Pena I, Caeiro V (1993) Preliminary note on the distribution and ecology of *Culicoides imicola* in Portugal. Medical and Veterinary Entomology 7: 23–26.

53. Rawlings P, Capela R, Pro MJ, Ortega MD, Pena I, et al. (1998) The relationship between climate and the distribution of *Culicoides imicola* in Iberia. Archives of Virology suppl. 14: 93–102.

54. Alahmed AM, Kheir SM, Al Khereiji MA (2010) Distribution of *Culicoides* Latreille (Diptera: Ceratopogonidae) in Saudi Arabia. Journal of Entomology 7: 227–234.

55. Hilali M, Abu-Elzein ELT, Al-Afaleq A, Mellor P, Boorman J, et al. (2003) *Culicoides* midges (Ceratopogonidae) in some localities of Saudi Arabia and their veterinary significance. Veterinarski Arhiv 73: 285–294.

56. De Meillon B (1959) Diptera (Nematocera) Ceratopogonidae. South African Animal Life 6: 325–355.

57. Labuschagne K, Gerber LJ, Espie I, Carpenter S (2007) *Culicoides* biting midges at the national zoological gardens of South Africa. Onderstepoort Journal of Veterinary Research 74: 343–347.

58. Linton Y-M, Mordue AJ, Cruickshank RH, Meiswinkel R, Mellor PS, et al. (2002) Phylogenetic analysis of the mitochondrial cytochrome oxidase subunit I gene of five species of the *Culicoides imicola* species complex. Medical and Veterinary Entomology 16: 139–146.

59. Meiswinkel R (1998) The 1996 outbreak of African horse sickness in South Africa - the entomological perspective. Archives of Virology suppl. 14: 69–83.

60. Nevill H, Nevill EM (1995) A survey of the *Culicoides* (Diptera: Ceratopogonidae) of the Umlalazi Nature Reserve in Zululand, South Africa, with notes on two species biting man. Onderstepoort Journal of Veterinary Research 62: 51–58.

61. Venter GJ, Graham SD, Hamblin C (2000) African horse sickness epidemiology: vector competence of South African *Culicoides* species for virus serotypes 3, 5 and 8. Medical and Veterinary Entomology 14: 245–250.

62. Venter GJ, Meiswinkel R, Nevill EM, Edwardes M (1996) *Culicoides* (Diptera:Ceratopogonidae) associated with livestock in the Onderstepoort area, Gauteng, South Africa as determined by light-trap collections. Onderstepoort Journal of Veterinary Research 63: 315–325.

63. Calvete C, Estrada R, Miranda MA, Borras D, Calvo JH, et al. (2008) Modelling the distributions and spatial coincidence of bluetongue vectors *Culicoides imicola* and the *Culicoides obsoletus* group throughout the Iberian peninsula. Medical and Veterinary Entomology 22: 124–134.

64. Gerry AC, Sarto I Monteys V, Moreno Vidal JO, Francino O, Mullens BA (2009) Biting rates of *Culicoides* midges (Diptera: Ceratopogonidae) on sheep in Northeastern Spain in relation to midge capture using UV light and carbon dioxide-baited traps. Journal of Medical Entomology 46: 615–624.

65. Goldarazena A, Romón P, Aduriz G, Balenghien T, Baldet T, et al. (2008) First record of *Culicoides imicola*, the main vector of bluetongue virus in Europe, in the Basque Country (northern Spain). Veterinary Record 162: 820–821.

66. Miranda MA, Borràs D, Rincón C, Alemany A (2003) Presence in the Balearic Islands (Spain) of the midges *Culicoides imicola* and *Culicoides obsoletus* group. Medical and Veterinary Entomology 17: 52–54.

67. Ortega MD, Holbrook FR (1994) Presence of *Culicoides imicola* (Diptera, Ceratopogonidae) In Jaen, Spain. Journal of the American Mosquito Control Association 10: 463–463.

68. Ortega MD, Lloyd JE, Holbrook FR (1997) Seasonal and geographical distribution of *Culicoides imicola* Kieffer (Diptera: Ceratopogonidae) in southwestern Spain. Journal of the American Mosquito Control Association 13: 227–232.

69. Ortega MD, Mellor PS, Rawlings P, Pro MJ (1998) The seasonal and geographical distribution of *Culicoides imicola*, *C. pulicaris* group and *C. obsoletus* group biting midges in central and southern Spain. Archives of Virology suppl. 14: 85–91.

70. Sarto I Monteys V, Ventura D, Pages N, Aranda C, Escosa R (2005) Expansion of *Culicoides imicola*, the main bluetongue virus vector in Europe, into Catalonia, Spain. Veterinary Record 156: 415–417.

71. Mellor PS, Osborne R, Jennings DM (1984) Isolation of bluetongue and related viruses from *Culicoides spp.* in the Sudan. Journal of Hygiene (Cambridge) 93: 621–628.

72. Mohammed MEH, Mellor PS (1990) Further studies on bluetongue and bluetongue-related Orbiviruses in the Sudan. Epidemiology and Infection 105: 619–632.

73. Al-Busaidy SM, Mellor PS (1991) Epidemiology of bluetongue and related orbiviruses in the Sultanate of Oman. Epidemiology and Infection 106: 167–178.

74. Taylor WP, Al Busaidy SM, Mellor PS (1991) Bluetongue in the sultanate of Oman, a preliminary epidemiological study. Epidemiology and infection 107: 87–97.

75. Cagienard A, Griot C, Mellor PS, Denison E, Stark KDC (2006) Bluetongue vector species of *Culicoides* in Switzerland. Medical and Veterinary Entomology 20: 239–247.

76. Chaker E, Sfari M, Rabhi M, Rouis M, Babba H, et al. (2005) Note faunistique sur les *Culicoides* (Diptera, Ceratopogonidae) du Gouvernorat de Monastir (Tunisie). Parasite 12: 359–361.

77. Hammami S, Bouzid M, Hammou F, Fakhfakh E, Delécolle JC (2008) Occurrence of *Culicoides spp.* (Diptera: Ceratopogonidae) in Tunisia, with emphasis on the bluetongue vector *Culicoides imicola*. Parasite 15: 179–181.

78. Dik B, Yağci S, Linton Y-M (2006) A review of species diversity and distribution of *Culicoides* Latreille, 1809 (Diptera: Ceratopogonidae) in Turkey. Journal of Natural History 40: 1947–1967.

79. Braverman Y, Phelps RJ (1981) Species composition and blood-meal identification in samples of *Culicoides* (Diptera: Ceratopogonidae) collected near Salisbury, Zimbabwe in 1976-77. Journal of the Entomological Society of Southern Africa 44: 315–323.

80. Musuka GN, Meiswinkel R, Baylis M, Kelly PJ, Mellor PS (2001) Prevalence of *Culicoides imicola* and other species (Diptera : Ceratopogonidae) at eight sites in Zimbabwe. Journal of the South African Veterinary Association-Tydskrif Van Die Suid-Afrikaanse Veterinêre Vereniging 72: 62–63.

81. Anderson RP, Gómez-Laverde M, Peterson AT (2002) Geographical distributions of spiny pocket mice in South America: insights from predictive models. Global Ecology and Biogeography 11: 131–141.

82. Webber BL, Yates CJ, Le Maitre DC, Scott JK, Kriticos DJ, et al. (2011) Modelling horses for novel climate courses: insights from projecting potential distributions of native and alien Australian acacias with correlative and mechanistic models. Diversity and Distributions 17: 978–1000.

83. Trabucco A, Zomer RJ, Bossio DA, Van Straaten O, Verchot LV (2008) Climate change mitigation through afforestation/reforestation : A global analysis of hydrologic impacts with four case studies. Agriculture, Ecosystems & Environment 126: 81–97.

84. Zomer RJ, Trabucco A, Bossio DA, Verchot LV (2008) Climate change mitigation : A spatial analysis of global land suitability for clean development mechanism afforestation and reforestation. Agriculture, Ecosystems & Environment 126: 67–80.

85. Siebert S, Döll P, Feick S, Frenken K, Hoogeveen J (2007) Global Map of Irrigation Areas version 4.0.1. University of Frankfurt and FAO, Rome, Italy.

86. Siebert S, Döll P, Hoogeveen J, Faures J-M, Frenken K, et al. (2005) Development and validation of the global map of irrigation areas. Hydrology and Earth System Sciences 9: 535–547.

87. Casati S, Racloz V, Delécolle JC, Kuhn M, Mathis A, et al. (2009) An investigation on the *Culicoides* species composition at seven sites in southern Switzerland. Medical and Veterinary Entomology 23: 93–98.

88. Pagès N, Muñoz-Muñoz F, Talavera S, Sarto V, Lorca C, et al. (2009) Identification of cryptic species of *Culicoides* (Diptera: Ceratopogonidae) in the subgenus *Culicoides* and development of species-specific PCR assays based on barcode regions. Veterinary Parasitology 165: 298–310.

89. Rawlings P, Capela R, Pro MJ, Ortega MD, Pena I, et al. (1998) The relationship between climate and the distribution of *Culicoides imicola* in Iberia. Archives of Virology suppl. 14: 93–102.

90. Lobo JM, Jimenez-Valverde A, Hortal J (2010) The uncertain nature of absences and their importance in species distribution modelling. Ecography 33: 103–114.
